# Value of inferior vena cava collapsibility index as marker of heart failure in chronic obstructive pulmonary disease exacerbation

**DOI:** 10.1186/s12872-023-03585-1

**Published:** 2023-11-24

**Authors:** Cyrine Kouraichi, Adel Sekma, Khaoula Bel Haj Ali, Ikram Chamtouri, Sarra Sassi, Marwa Toumia, Hajer Yaakoubi, Rym Youssef, Mohamed Amine Msolli, Kaouthar Beltaief, Zied Mezgar, Mariem Khrouf, Wahid Bouida, Zohra Dridi, Riadh Boukef, Hamdi Boubaker, Mohamed Habib Grissa, Semir Nouira

**Affiliations:** 1 Research Laboratory LR12SP18, Monastir University, 5000 Monastir, Tunisia; 2 Emergency Department, Fattouma Bourguiba University Hospital, 5000 Monastir, Tunisia; 3 Cardiology Department, Fattouma Bourguiba University Hospital, 5000 Monastir, Tunisia; 4 Emergency Department, Regional Hospital Ksar Helal, Monastir, Tunisia; 5 Emergency Department, Sahloul University Hospital, 4011 Sousse, Tunisia; 6 Emergency Department, Farhat Hached University Hospital, 4031 Sousse, Tunisia; 7 Emergency Department and Laboratory Research (LR12SP18), Fattouma Bourguiba University Hospital, 5000 Monastir, Tunisia

**Keywords:** Heart failure, Dyspnea, Emergency AECOPD, Ultrasound, Collabsibility index

## Abstract

**Introduction:**

Inferior vena cava (IVC) diameter variability with respiration measured by ultrasound was found to be useful for the diagnosis of heart failure (HF) in ED patients with acute dyspnea. Its value in identifying HF in acute exacerbation of c*hronic obstructive pulmonary disease* exacerbation (AECOPD) was not specifically demonstrated.

**Objective:**

To determine the value of ΔIVC in the diagnosis of HF patients with AECOPD.

**Methods:**

This is a prospective study conducted in the ED of three Tunisian university hospitals including patients with AECOPD. During this period, 401 patients met the inclusion criteria. The final diagnosis of HF is based on the opinion of two emergency experts after consulting the data from clinical examination, cardiac echocardiography, and BNP level. The ΔIVC was calculated by two experienced emergency physicians who were blinded from the patient’s clinical and laboratory data. A cut off of 15% was used to define the presence (< 15%) or absence of HF (≥ 15%). Left ventricular ejection fraction (LVEF) was also measured. The area under the ROC curve, sensitivity, specificity, and positive and negative predictive values were calculated to determine the diagnostic and predictive accuracy of the ΔIVC in predicting HF.

**Results:**

The study population included 401 patients with AECOPD, mean age 67.2 years with male (68.9%) predominance. HF was diagnosed in 165 (41.1%) patients (HF group) and in 236 patients (58.9%) HF was excluded (non HF group). The assessment of the performance of the ΔIVC in the diagnosis of HF showed a sensitivity of 37.4% and a specificity of 89.7% using the threshold of 15%. The positive predictive value was 70.9% and the negative predictive value was 66.7%. The area under the ROC curve was 0.71(95%, CI 0.65–0.76). ΔIVC values were not different between HF patients with reduced LVEF and those with preserved LVEF.

**Conclusion:**

Our results showed that ΔIVC has a good value for ruling out HF in ED patients consulting for AECOPD.

## Introduction

Chronic obstructive pulmonary disease (COPD) is a heterogeneous lung condition characterized by chronic respiratory symptoms (dyspnea, cough, sputum production) due to abnormalities of the airways (bronchitis, bronchiolitis) and/or alveoli (emphysema) that cause persistent, often progressive, airflow obstruction [1]. It is an increasingly common disease and a major public health problem. According to the latest Global Initiative for Obstructive Lung Disease report, COPD is the fourth leading cause of death in the world [1]. It resulted in more than 3 million deaths in 2019 [1]. The incidence of COPD is 3.7% in Tunisians aged 40 years and over [2]. The evolution of COPD is marked by recurrent life-threatening exacerbations that increase the deterioration of respiratory function and progression to chronic respiratory failure [3, 4]. Consequently, any exacerbation must be treated promptly and adequately. This requires a good identification of the triggering factor for immediate and targeted etiological treatment [5, 6]. Among the commonest etiologic factors of acute exacerbation of COPD (AECOPD) is heart failure (HF) and the association of COPD with cardiovascular comorbidities is frequent. More than 20% of AECOPD are associated with HF [4], but this association is thought to be underestimated as the available diagnostic tools to identify HF in COPD patients consulting for dyspnea lack specificity [7, 8]. The gold standard in the diagnosis of HF is cardiac ultrasound and brain natriuretic peptide (BNP) testing [9, 10]; but these methods are problematic in terms of their availability in the emergency department (ED) and the need for an experienced sonographer. Ultrasound of the inferior vena cava (IVC) is an easy, convenient, and validated examination for the diagnosis of HF by measuring the collapsibility index (ΔIVC) [11–14]. Indeed, the IVC is a compliant vessel whose diameter change during respiration cycle. Changes of IVC diameter are generally accentuated when the IVC intravascular pressure is low, and they decrease when IVC is congested. This feature is the basis for the diagnosis of HF, which is characterized by a decrease in the respiratory IVC diameter variation [15]. COPD is associated with structural changes in the pulmonary vessels and right heart dysfunction classically referred as cor pulmonale. These conditions increase IVC congestion and reduce IVC inspiratory collapse even though there is no left HF. Consequently, it is expected that the specificity of ΔIVC in the diagnosis of HF will decrease in AECOPD. Thus, the aim of our study was to assess the value of ΔVCI in the diagnosis of HF in AECOPD in the emergency department for acute exacerbation.

## Patients and methods

### Study design

This is a cross sectional prospective study conducted in the ED of three Tunisian university hospitals: Fattouma Bourguiba Monastir, Sahloul Sousse, and Farhat Hached Sousse from January 2022 to March 2022.

### Study population

Inclusion criteria: patients aged more than 18 years, and consulting the ED for acute dyspnea with a final diagnosis of AECOPD were included. AECOPD is defined as an event characterized by dyspnea and/or cough and sputum that worsens in < 14 days and is often associated with increased local and systemic inflammation caused by airway infection, pollution, or other insult to the lungs [1]. Exclusion criteria: Patients with hemodynamic instability (presence of peripheral signs of shock, use of vasoactive drugs) or respiratory distress, use of mechanical ventilation, and/or with altered consciousness (Glasgow Coma Score ≤ 13) were excluded. Similarly, patients not consenting to the protocol were excluded.

The study was carried out in accordance with declaration of Helsinki and was approved by the Fattouma Bourguiba ethic committee; informed consent was obtained before the start of the protocol in all included patients. The study was registered in the ClinicaTrials.gov register under the number NCT05327374 (date of first registration: 01/03/2022).

### Data collection

After the consent of included patients, data from the clinical examination and complementary examinations were collected. A systematic collection of the following clinical data was performed including age, sex, body mass index (BMI), cardiovascular risk factors such as hypertension, diabetes, dyslipidemia, smoking, HF, and baseline New York Heart Association (NYHA) dyspnea stage (Table 1). For all included patients, data on physical examination, electrocardiogram, standard biological tests, BNP level, and cardiac ultrasound data were collected. Cardiac ultrasound is performed using a 5-MHz convex probe device (Sonsonite Inc, Bothell, WA). Two experienced emergency physicians performed IVC diameter measurements during the study. For each patient only one rater was performed. Evaluation was performed with the patient lying supine if tolerated or in a semi recumbent position with the head-of-bed elevated to 30°. The anteroposterior diameter of the IVC was measured at its maximum diameter during expiration and its minimal diameter during inspiration by TM-mode at the subxiphoid region proximal to the confluence of the hepatic veins. Measurements were averaged over 3 respiratory cycles to account for variations in respiratory efforts. The IVC collapsibility index is calculated by the following formula: ΔIVC = (max IVC diameter—min IVC diameter)/max IVC diameter. Measurements of the IVC was obtained during passive respiration. A ΔIVC < 15% is retained to define the presence of HF. The left ventricular ejection fraction (LVEF) is also measured (cut-off preserved/reduced). The operator was unaware of the patient's clinical and laboratory data. The final diagnosis of HF is based on the opinion of two emergency experts after consulting the data from the clinical examination, cardiac echocardiography, and BNP level according to the ESC 2021 guidelines [8].

**Table 1:** Baseline patients’ characteristics

	**Heart Failure** * n* = 165 (41.1%)	**Non- Heart Failure** ***n* = 236 (58.9%)**	**Overall population**
*Age (years), mean (SD)*	70 (10)	64(12)	67,2(12,2)
*Sex-ratio*	1.75	2. 63	2.2
*Comorbidities, n (%)*			
*Chronic heart failure*	51(30.9)	11(4.6)	62(15.5)
*Coronary artery disease,*	32(19.3)	21(8.8)	53(13.2)
*Hypertension*	92(55.7)	74(31.3)	166(41.4)
*Diabetes*	70(42.4)	67(28.3)	137(34.2)
*Chronic kidney disease, n (%)*	20(14.7)	10(5.6)	30(7.5)
*NYHA classification, n(%)*			
*I*	1(0.6)	11(4.6)	12(3.7)
*II*	26(15.7)	62(26.2)	88(27.1)
*III*	62(37,5)	81(34.2)	143(44)
*IV*	37(22.4)	45(19.1)	82(25.2)
*Fever, n (%)*	26 (15.7)	45 (19.1)	71(17.7)
*Systolic blood pressure (mmHg), mean (SD)*	136(21,5)	139(31,7)	137.8(57.1)
*Diastolic blood pressure (mmHg), mean (SD)*	73.15 (15.7)	76.17 (23.2)	74.9(18.5)
*Orthopnea, n(%)*	42(25.4)	57(24.1)	99(24.9)
*Respiratory rate (cycle/min), mean (SD)*	28.5 (9.7)	27.12 (7.9)	27(9.7)
*cardiac frequency (bpm), mean (SD)*	102.1 (24.5)	106.2 (22.4)	104.4(38.7)
*Atrial fibrillation, n(%)*	46(27.9)	35(14.8)	81(20.2)
*pH, mean (SD)*	7.35 (1.03)	7.35 (0.08)	7.36(0.07)
*PaCO*_*2*_* (KPa), mean (SD)*	6.8 (6.5)	7.6 (5.6)	7.3(6.4)
*PaO*_*2*_* (KPa), mean (SD)*	11.5 (8.8)	12 (7.2)	11.8(8.7)
*HCO*_*3*_^*−*^*(mmol/l), mean (SD)*	25.8 (8.3)	27 (9.2)	26.9(10.8)
*SaO*_*2*_* (%), mean (SD)*	89.8 (7.1)	89.7 (10.2)	89.8(12.8)
*BNP (pg/ml), median [ IQR]*	306 [172–672]	69 [29–154]	165[58–432]

*Abbreviation*: *NYHA* New York Heart Association, *bpm* beat per minute, *BNP* Brain Natriuretic Peptide, *IQR* Interquartile Range

### Statistical analysis

Qualitative data were presented as numbers and frequencies (%). Comparisons between categorical variables were made using Pearson's chi-2 test. For quantitative variables, normality was assessed using the Kolmogorov–Smirnov test. Continuous variables were expressed as mean (DS) or median (IQR), as appropriate. Quantitative variables were compared using a student’s t-testor non-parametric tests as appropriate. Sensitivity, specificity, positive and negative predictive values, likelihood ratios, and the receiver operating characteristic (ROC) of ΔIVC for determining the diagnosis of HF were calculated with the corresponding 95% CIs with a test of significance set at *P* < 0.05. The results obtained in this study were analyzed using SPSS statistical software (English version 22, IBM Corp., Armonk, NY).

## Results

**Fig. 1:**
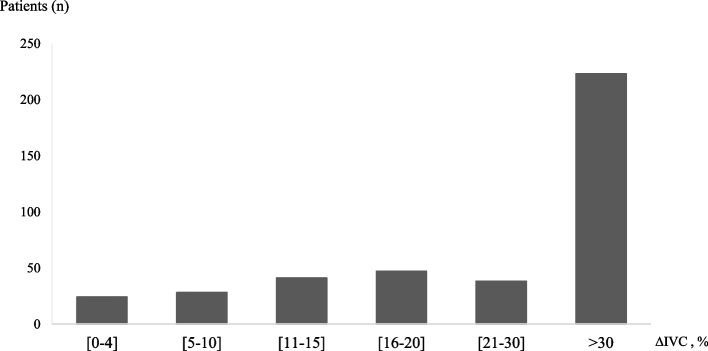
Distribution of patients according to inferior vena cava collapsibility index (*ΔIVC)* value intervals. *Abbreviations: n (number)*

**Fig. 2:**
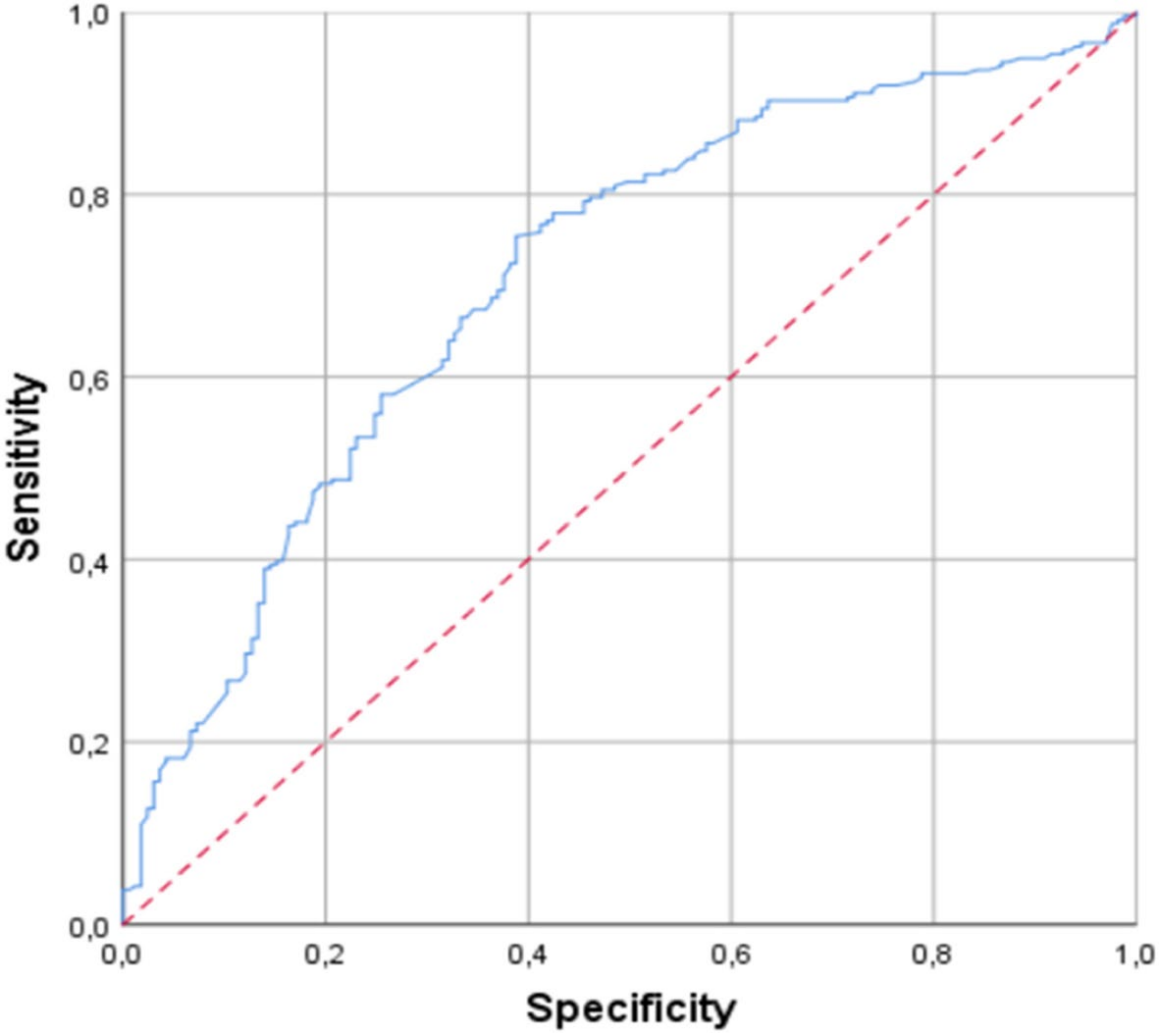
Receiver operating characteristic curve of inferior vena cava collapsibility index (ΔIVC) in the diagnosis of heart failure

During the study period, 431 patients with AECOPD were included. Thirty patients (6.9%) were excluded because of the inability to visualize the IVC. The patients were divided into two groups according to the final diagnosis of HF; 165 patients (41.1%) had a final diagnosis of HF (HF group) and 236 patients (58.9%) without HF (non HF group). The characteristics of the patients are summarized in Table 1. The mean age of the population was 67.2 ± 12.2 years; hypertension was the most common cardiovascular risk factor (41.4%), 34.2% of patients had diabetes, and 15.5% had history of chronic HF (CHF). Patients in the HF group had more comorbidities with higher rates of hypertension, CHF, coronary artery disease, and diabetes. The mean LVEF was 40.2% in HF patients and 63.2% in non-HF patients. 65 patients (39.4%) in the HF group had a preserved LVEF. The mean ΔIVC was 20.5% ± 5.1% in the HF group, and 35.2% ± 6.5% in the non-HF group (*p* < 0.001). The mean ΔIVC in the HF subgroup with reduced LVEF (LVrEF) was 20.2% ± 6.1%, and 20.8% ± 7.3% in the HF subgroup with preserved LVEF (LVpEF). The distribution of patients according to ΔIVC value intervals is shown in Fig. 1. Almost half of patients (47.4%) had ΔIVC > 30%. Table 2 shows the diagnostic performance of ΔIVC using different thresholds. The intraoberserver reliability was fair with a Kappa index of 0.28; the interobserver reliability was not assessed. For a threshold of 15% which appears to be associated with the best diagnostic performance, the sensitivity and specificity of ΔIVC were 37.4% and 89.7% respectively; the positive predictive value is 70.9% and the negative predictive value is 66.7%. The area under the ROC curve is 0.71(95%, CI 0.65 – 0.76) (Fig. 2).

**Table 2:** Performance of inferior vena cava collapsibility index in the diagnosis of heart failure with different thresholds

ΔIVC (%)	Se (%)	Sp (%)	PPV (%)	NPV (%)	LR +	LR -
5	4.9 [2.8–7]	96.5 [94.7–8.3]	50.1 [45.2–55]	59.2 [54.4–64]	1.4	0.9
10	17.4 [13.7–21.1]	94.4 [92.1–6.7]	68.5 [64–73]	62 [57.2–66.8]	3.1	0.8
15	37.5 [2, 8–42]	89.7 [86.7–2.7]	70.9 [66.5–5.3]	66.7 [62.1–71.3]	3.5	0.7
20	50.3 [45.4–55.2]	81.9 [78.1–5.7]	66.1 [61.5–0.7]	70.2 [65.7–74.7]	2.7	0.6

*Abbreviations*: *Se* Sensitivity, *Sp* Specificity, *PPV* Positive predictive value, *NPV* Negative predictive value, *LR* Likelihood-Ratio, *ΔIVC* inferior vena cava collapsibility index

## Discussion

This study showed that discriminatory power of ΔIVC in the diagnosis of HF in AECOPD is acceptable. The ΔIVC has good specificity but low sensitivity.

Patients with COPD are at high risk for cardiovascular disease, including HF [12]. The prevalence of congestive HF in COPD patients in different series ranges from 7 to 30% [13]. The diagnosis of one of these conditions may mask the other [14, 15] and this combination presents many diagnostic and therapeutic dilemmas17 [16]. Importantly, HF is often undetected in patients with AECOPD [16, 17]. Identifying HF in AECOPD in a rapid and non-invasive manner is very important in the ED. BNP is increasingly used in clinical practice as a marker of HF but lacks specificity in many clinical situations. In particular, BNP levels of up to 500 pg/mL may be observed in cases of right ventricular dilatation [18–20]. Tung et al. showed that in COPD patients with a history of HF, the specificity of BNP is only 47% [21]. To better identify HF, a more efficient test is needed. Based on its usefulness for the detection of hemodynamic congestion, IVC ultrasonography has been recently proposed in HF diagnosis. The IVC is a compliant blood vessel subject to extramural pressure and its caliber varies with respiration [22], blood volume [23], and right heart function [24]. In cases of congestive HF, volume overload dilates the IVC to the limits of its elasticity such that the increase in pressure during expiration leads to a minimal increase in diameter. Previous studies have shown that changes in IVC diameter correlate with ventricular filling pressures [25, 26]. In patients with chronic HF referred for a right heart catheterization, IVC diameter performed the best among several indexes (area under the receiver operating characteristic curve, 0.89) at identifying those with pulmonary capillary wedge pressures ≥ 15 mm Hg [27]. ΔVCI measurement in patients with acute undifferentiated dyspnea was shown to provide a good diagnostic approach in the ED. Blehar et al*.* reported a sensitivity of 0.93 and a specificity of 0.84 for detecting HF in 14 out of 46 patients for a ΔVCI < 15% [28]. Anderson et al*.* reported a sensitivity of 0.52 and a specificity of 0.86 for detecting HF in 44 out of 101 patients for a ΔIVC < 20% [29]. Yamanoǧlu et al*.* reported a sensitivity of 0.84 and a specificity of 0.92 for detecting HF using a ΔVCI cut-off < 52% [30]. Miller et al. reported a sensitivity of 0.80, and a specificity of 0.81 for detecting HF in 35 out of 89 patients for a ΔIVC < 33% [31]. So, except the study of Anderson et al., all the cited studies reported a good sensitivity and specificity with variable thresholds. In this study, we confirmed the good specificity of ΔIVC contrary to what one might expect in patients with a prevalence of pulmonary hypertension and cor pulmonale that can exceed 50% [32]. Different cut-offs of delta IVC index were assessed and the best cut-off associated with the highest specificity with an acceptable sensibility was 15% as the one used in Blehar et al. study. Reduced right ventricular compliance and/or increased filling pressures in COPD patients, especially during acute exacerbation, expose the right atrium to pressure load and dilatation and this should decrease respiratory IVC diameter changes. The fact that mean ΔIVC is 24% in our HF patients means that IVC collapsibility was not altered in our AECOPD patients. To our opinion, these results emerge from 3 main causes. The first cause is probably related to the possibility that our patients were not severe enough to have a significant elevation of pulmonary artery pressure and systemic venous congestion. The second cause is related to the respiratory system mechanics of AECOPD patients which are characterized by the development of dynamic hyperinflation and intrinsic positive end-expiratory pressure or PEEPi [33]. Initiation of inspiratory flow requires inspiratory force to overcome PEEPi, which translates into an increased inspiratory effort during the triggering phase and generates high variations of intrathoracic pressure. These variations could be amplified by a simultaneous rise in intra-abdominal pressure making the IVC more easily compressible. The third reason is related to the location of IVC diameter measurement and probe orientation as variations of IVC diameters are significantly lower when recorded close to the right atria.

First, ΔIVC has a good specificity for the diagnosis of HF among patients with AECOPD but with weak sensibility. So ΔIVC may be helpful to rule-in the diagnosis of HF in AECOPD, but it cannot itself exclude it. So the ΔIVC may be helpful to rule-in the diagnosis of HF in AECOPD but it cannot exclude it. Second, this study was limited by its smaller size and the possible selection bias from the convenience sampling methodology. Our results could not be extrapolated to all AECOPD because severe patients were excluded. Third, we did not assess the reproducibility of the ΔIVC because we assumed that its reproducibility is generally good. Fourth, the blinded nature of the study may not be fully respected, but all provisions were made to reduce this bias. Fifth, we did not measure the direct impact of valvular diseases on IVC diameters, such as in the case of tricuspid or mitral regurgitation. This fact may affect sensitivity, but not specificity. Finally, the ΔVCI was measured after a lag time, approximately 4 h following ED admission (methods), a period during which the patient condition could be improved by treatment. This would be responsible for a decrease in the sensitivity of the ΔIVC in the diagnosis of HF.

## Conclusion

In summary, there are no studies that have specifically sought for assessment of IVC collapsibility index performance in the diagnosis of HF in AECOPD. Our results suggest that IVC collapsibility may still be considered in the diagnostic approach of HF in AECOPD patients, at least as a ruling-out test. In patients with ΔIVC value > 15%, HF cannot be excluded, while patients with ΔIVC under 15% are more likely to have HF. Further studies are needed to better objectify its diagnostic performance alone or in combination with other markers of HF.

## Data Availability

The datasets used and/or analysed during the current study are available from the corresponding author on reasonable request.
